# Clinical Characteristics of 19 Patients With Acid Sphingomyelinase Deficiency: A Case Series From Multiple Centers in Argentina

**DOI:** 10.1002/jmd2.70104

**Published:** 2026-06-28

**Authors:** Maria Cristina Robin, Consuelo Durand, Guillermo Guelbert, Norberto Guelbert, Mariana Leguizamon, Mariana Nuñez Miñana, Maria Alejandra Cédola, Maria Gabriela de Lourdes Pacheco, Evangelina Gonzalez, Marisa Spallina, Sandra Quijano

**Affiliations:** ^1^ Clínica Universitaria Reina Fabiola Córdoba Argentina; ^2^ Hospital Zonal Frías Frías Santiago del Estero Argentina; ^3^ Hospital Británico de Buenos Aires Buenos Aires Argentina; ^4^ Department of Neuropediatrics Fleni Buenos Aires Argentina; ^5^ CEMPE Buenos Aires Argentina; ^6^ Hospital de Niños de la Santísima Trinidad Córdoba Argentina; ^7^ Hospital del Niño Jesús San Miguel de Tucumán Argentina; ^8^ Hospital de Niños Sor María Ludovica La Plata Buenos Aires Argentina; ^9^ Clínica San Lucas Neuquén Argentina; ^10^ Hospital Público Materno Infantil Salta Argentina; ^11^ Sanatorio de Niños Rosario, Santa Fe Argentina; ^12^ Private Practice, Baradero Buenos Aires Argentina; ^13^ Hospital Julio C. Perrando Resistencia Chaco Argentina

**Keywords:** acid sphingomyelinase deficiency, ASMD, diagnosis, Niemann‐Pick disease Types A/B

## Abstract

Acid sphingomyelinase deficiency (ASMD), historically known as Niemann‐Pick disease, is a rare and potentially fatal lysosomal storage disease caused by pathogenic variants in the sphingomyelin phosphodiesterase 1 (*SMPD1*) gene, which encodes acid sphingomyelinase (ASM). Deficient ASM activity results in dysregulation of cellular membrane homeostasis and accumulation of sphingomyelin in multiple organs. ASMD spans a broad clinical spectrum, with symptoms varying at presentation depending on age at onset and degree and type of organ/systemic involvement. To describe the diagnostic experience and clinical manifestations of ASMD in Argentina, a national retrospective case series was conducted across seven centers in patients diagnosed between 1988 and 2022. Diagnosis was confirmed by reduced ASM activity, with *SMPD1* sequencing performed when possible. Nineteen patients (8 females/11 males; 0–76 years) were identified: Type A (4, 21%), Type B (12, 63%), Type A/B (2, 11%), and one unknown. Average age at symptom onset was 3.9 years, and average age at diagnosis was 11.4 years, corresponding to a diagnostic delay of 7.5 years. All patients presented with hepatosplenomegaly. Anemia (79%), pulmonary involvement (79%), thrombocytopenia (79%), and osteopenia (56%) were also reported in a majority of patients. Two patients were initially misdiagnosed with Gaucher disease. This series highlights the variety of clinical presentations and substantial diagnostic delays associated with ASMD in Argentina. Increasing awareness across specialties is essential to improve disease recognition, reduce time to diagnosis, and prevent misdiagnosis.

## Synopsis

This national retrospective case series in Argentina highlights the heterogeneous clinical presentation and prolonged diagnostic delays associated with ASMD, reinforcing the urgent need for increased clinician awareness to improve recognition and diagnosis.

**Abbreviations**
ASM: acid sphingomyelinase
ASMD: acid sphingomyelinase deficiency
*SMPD1*
: sphingomyelin phosphodiesterase 1

## 1 Introduction

Acid sphingomyelinase deficiency (ASMD), historically known as Niemann‐Pick disease Types A and B, is a rare and potentially fatal lysosomal storage disease [1, 2, 3, 4]. ASMD results from pathogenic variants in the gene *SMPD1* (sphingomyelin phosphodiesterase 1), which encodes for acid sphingomyelinase (ASM) [2, 3, 4]. Deficiency of ASM disrupts cellular membrane homeostasis and the accumulation of sphingomyelin and other sphingolipids in the cells of multiple organs, including the spleen, liver, lungs, bone marrow, lymph nodes, and in more severe forms, the central nervous system and peripheral nervous system [1, 3]. ASMD spans a broad spectrum of disease, with symptoms varying at presentation depending on age at onset and the degree and type of organ and systemic involvement. Disease manifestations vary but often include hepatosplenomegaly, liver dysfunction, interstitial lung disease, dyslipidemia, bleeding, and growth impairment [1, 4, 5]. Diagnosis of ASMD can be delayed months or years because of the heterogeneity of the presenting symptoms and misdiagnosis [6, 7]. Hepatosplenomegaly is the most common clinical presentation of ASMD, and differential diagnoses include chronic hepatitis B, hepatic malignancies, and other lysosomal storage diseases such as Gaucher disease [6, 8]. A definitive ASMD diagnosis is based on clinical evaluation and ASM enzymatic activity testing in leukocytes or fibroblasts [4, 5]. Diagnosis can be confirmed by genetic sequencing [3, 4].

Traditionally, ASMD is divided into three subtypes based on clinical assessment and disease course: infantile neurovisceral (Type A), chronic visceral (Type B), and chronic neurovisceral (Type A/B). The most severe form of ASMD is Type A, with symptoms beginning in infancy and rapidly progressing neurodegeneration, typically leading to death by 3 years of age. ASMD Type B is characterized by little or no neurological involvement, with symptom onset ranging from infancy to adulthood. Patients with ASMD Type B may have a normal lifespan but can die prematurely, most commonly from respiratory and/or liver failure. An intermediate subtype, ASMD Type A/B, falls in the middle of the disease spectrum, with neurological involvement that is variable in onset and severity and progresses more slowly than the neurodegeneration observed in Type A. ASMD Type A/B may result in mortality in childhood or adulthood [3].

The global incidence of ASMD is estimated at 0.4 to 0.6 in 100 000 live births annually [9], although this is likely an underestimation because of the rarity of ASMD and limited awareness among health care providers [1, 5, 8]. This case series aims to increase recognition of ASMD by describing the diagnostic experience and clinical manifestations in 19 patients with ASMD across 7 centers in Argentina.

## 2 Methods

This national retrospective case series was conducted across multiple centers in Argentina and was approved by the Training, Teaching, and Research Committee of the Ministry of Health of the Province of Santiago del Estero, Argentina. Informed consent was obtained from adult patients or from the parents or legal guardians of pediatric patients when required. For deceased patients and due to the retrospective nature of the study, consent requirements were waived in accordance with local regulations. All data were anonymized before analysis.

Patients were identified retrospectively at each participating center based on a confirmed diagnosis of ASMD. Consecutive cases were included when available. Data were collected from medical records using a standardized approach across centers. Collected variables included demographic data, clinical presentation, laboratory findings, and disease course. Information on consanguinity and family history was obtained from clinical records as part of routine patient history.

Patients included in this case series were diagnosed with ASMD between 1988 and 2022 according to established international criteria [6, 8]. Patients were classified into ASMD subtypes (Types A, B, and A/B) based on clinical criteria, including age at symptom onset, presence or absence of neurological involvement, and overall disease course. Type A was defined by early onset disease with severe and progressive neurodegeneration; Type B by the absence of neurological involvement and a predominantly visceral phenotype; and Type A/B by an intermediate phenotype with variable neurological involvement. Diagnosis was confirmed by demonstrating reduced ASM activity, measured using isolated leukocytes or dried blood spot testing [1, 10, 11]. Genetic confirmation by *SMPD1* sequencing was performed when available. Pulmonary involvement was defined based on clinical findings and imaging studies, including chest X‐ray and/or computed tomography, supported by pulmonary function tests (e.g., spirometry and diffusing capacity for carbon monoxide) when available. Differential diagnosis with Gaucher disease was established through measurement of specific enzyme activities, including acid sphingomyelinase and β‐glucocerebrosidase, supported by biomarker analysis when available, and confirmed by molecular testing of the *SMPD1* and *GBA* genes when performed. Missing data were handled descriptively and were not imputed. The primary aim of data collection was to assess the diagnostic experience and clinical characteristics of these patients.

## 3 Results

All 19 patients described in this case series were from Argentina. Eight patients were female and 11 were male, ranging in age from 0 to 76 years. Of the 19 patients, ASMD Type A was identified in four patients (21%), ASMD Type B in 12 patients (63%), and ASMD Type A/B in two patients (11%). One patient could not be definitively classified into a specific ASMD subtype due to incomplete clinical data, particularly regarding neurological involvement and disease progression. The average age at symptom onset for all patients was 3.9 years (range: birth to 18.6 years; Table 1). When analyzed by subtype, the average age at symptom onset was 4 months (range: birth to 6 months) for ASMD Type A, 5.8 years (range: 9 months to 18.6 years) for ASMD Type B, and 1.8 years (range: 6 months to 3 years) for Type A/B. The overall average age at diagnosis was 11.4 years (range: 1 month to 49 years; Table 1). By subtype, the average age at diagnosis was 7 months (range: 1 month to 1 year) for patients with ASMD Type A, 16.8 years (range: 1.5 years to 49 years) for Type B, and 4.3 years (range: 6 months to 8 years) for Type A/B. The median delay from the initial presentation of symptoms to the definitive diagnosis of ASMD was 0.75 years (mean: 7 years; range: 0 to 35 years) for all patients and 3.5 months (mean: 3.3 months, range: 0 months to 6 months), 2.9 years (mean: 11.6 years, range: 0 months to 35 years), and 2.5 years (mean: 2.5 years, range: 0 months to 5 years) for patients with ASMD subtypes A, B, and A/B, respectively. Before receiving a definitive diagnosis, patients consulted an average of 3 (range: 0 to 5) specialties. Two siblings in the cohort with ASMD Type B were originally misdiagnosed with Gaucher disease. Gaucher disease was considered a differential diagnosis for all patients.

**TABLE 1:** Initial symptom onset, time to diagnosis, and specialists consulted.

ASMD subtype	Patient	Age at symptom onset	First symptoms	Age at diagnosis	Age at death	Misdiagnosis	Specialists consulted
A	1	6 months	—	1 year	2 years 2 months	—	Pediatrics, hematology, neurology
	2	6 months	Hepatosplenomegaly, globular abdomen, pancytopenia	1 year	2 years 3 months	—	Pediatrics, hematology
	3	6 months	Anemia	1 month	3 years 6 months	—	—
	4	At birth	Anemia	1 month	4 years 3 months	—	—
B	5	5 years	Chronic diarrhea	40 years	NA	—	Pediatrics, hematology, gastroenterology, nutrition
	6	16 years 9 m	Hepatosplenomegaly, anemia	47 years	NA	Gaucher disease	Hematology, gastroenterology, pulmonology, trauma
	7	18 years 7 m	Hepatosplenomegaly, anemia	49 years	68 years	Gaucher disease	Hematology, gastroenterology, pulmonology, trauma
	8	1 year 5 m	Anemia, globular abdomen	5 years	NA	—	Pediatrics, hematology, gastroenterology
	9	2 years	Anemia, low weight	22 years	NA	—	Pediatrics, gynecology
	10	3 years 1 m	Hepatosplenomegaly	3 years 7 months	NA	—	Pediatrics, gastroenterology
	11	—	—	15 years	18 years	—	Pediatrics, hematology, pulmonology
	12	2 years 9 m	Hepatosplenomegaly, globular abdomen	3 years 9 months	NA	—	Pediatrics, hematology, gastroenterology
	13	1 year	Hepatosplenomegaly, globular abdomen	3 years 3 months	NA	—	Pediatrics, hepatology, neurology
	14	2 years	Hepatosplenomegaly	2 years 5 months	NA	—	—
	15	10 years	Hepatosplenomegaly	10 years	NA	—	Pediatrics, hematology, pulmonology
	16	9 months	—	1 year 6 months	NA	—	Pediatrics, hematology, hepatology, gastroenterology, pulmonology
A/B	17	3 years	Hepatosplenomegaly	8 years	NA	—	Pediatrics, hematology, gastroenterology
	18	6 months	Hepatosplenomegaly, anemia	6 months	2 years 8 months	—	Pediatrics
—	19	2 years	Hepatosplenomegaly, anemia	2 years 6 months	13 years	—	Pulmonology, hepatology, neurology, ophthalmology

*Note:* – denotes data not available. Shaded gray rows indicate patient is deceased.

Abbreviations: ASMD, acid sphingomyelinase deficiency; NA, not applicable.

Hepatosplenomegaly was the first symptom reported in the majority of patients (11/19, 58%). Other initial presenting symptoms observed included anemia (8/19, 42%), globular abdomen (4/19, 21%), low weight (1/19, 5%), chronic diarrhea (1/19, 5%), and pancytopenia (1/19, 5%). More broadly, the most common clinical manifestations observed over the course of patient history included hepatosplenomegaly (19/19, 100%), anemia (15/19, 79%), pulmonary involvement (15/19, 79%), thrombocytopenia (15/19, 79%), osteopenia (9/16, 56%), leukopenia (8/19, 42%), neurological involvement (6/19, 32%), neurological symptoms (6/19, 32%), and cherry‐red spot (5/19, 26%; Table 2).

**TABLE 2:** Clinical features of patients with ASMD included in this cohort.

ASMD subtype	Patient	Hepato‐spleno‐megaly	Anemia	Pulmonary involvement	Thrombo‐cytopenia	Osteo‐penia	Leukopenia	Neurological involvement^a^	Cherry‐red spot
A	1	Yes	Yes	Yes	Yes	Yes	No	Developmental delay and hypotonia	No
	2	Yes	Yes	Yes	Yes	No	Yes	Developmental delay and hypotonia	Yes
	3	Yes	Yes	Yes	Yes	No	Yes	Hypotonia	Yes
	4	Yes	Yes	Yes	Yes	No	Yes	Hypotonia	Yes
	Total *n*/*N*, %	4/4, 100%	4/4, 100%	4/4, 100%	4/4, 100%	1/4, 25%	3/4, 75%	4/4, 100%	3/4, 75%
B	5	Yes	No	Yes	No	No	No	No	Yes
	6	Yes	Yes	Yes	Yes	Yes	No	No	No
	7	Yes	Yes	Yes	Yes	Yes	No	No	No
	8	Yes	Yes	Yes	Yes	Yes	No	No	No
	9	Yes	Yes	No	Yes	No	Yes	No	No
	10	Yes	No	No	No	Yes	No	No	No
	11	Yes	Yes	Yes	Yes	—	No	No	No
	12	Yes	No	Yes	No	No	No	No	No
	13	Yes	Yes	No	Yes	No	No	No	No
	14	Yes	Yes	No	Yes	Yes	Yes	No	No
	15	Yes	No	Yes	Yes	—	Yes	No	No
	16	Yes	Yes	Yes	Yes	Yes	No	No	No
	Total *n*/*N*, %	12/12, 100%	8/12, 67%	8/12, 67%	9/12, 75%	6/10, 60%	3/12, 25%	0/12, 0%	1/12, 8%
A/B	17	Yes	Yes	Yes	Yes	Yes	Yes	Hypotonia, ataxia, and neurocognitive impairment; onset of neurological involvement at 20 years	No
	18	Yes	Yes	Yes	No	—	No	Hypotonia, ataxia, and neurocognitive impairment; onset of neurological involvement at 5 months	No
	Total *n*/*N*, %	2/2, 100%	2/2, 100%	2/2, 100%	1/2, 50%	1/1, 100%	1/2, 50%	2/2, 100%	0/2, 0%
—	19	Yes	Yes	Yes	Yes	Yes	Yes	No	Yes
Total *n*/*N*, %		19/19, 100%	15/19, 79%	15/19, 79%	15/19, 79%	9/16, 56%	8/19, 42%	6/19, 32%	5/19, 26%

*Note:* – denotes data not available. Shaded gray rows indicate patient is deceased.

Abbreviation: ASMD, acid sphingomyelinase deficiency.

^a^ Neurological involvement was defined as ASMD‐related neurological disease. Two patients with ASMD Type B had non‐ASMD‐related neurological symptoms.

Patients included in this case series resided in multiple provinces in Argentina, as shown in Figure S1 and Table 3. Consanguinity was not observed in this patient cohort, although a positive family history of ASMD was documented in 6 patients (Table 3), each belonging to 1 of 3 sibling pairs included in this cohort. Genetic sequencing of the *SMPD1* gene was performed in nine patients. Among these, 14 pathogenic variants were observed: c.1426C>T was observed in three patients, c.1160G>A was observed in two patients, and c.1327C>T, c.1801G>A, c.96G>A, c.1107_1110dup, c.1492C>T, c.319‐2A>G, c.1406A>G, c.1553C>T, c.518dup, c.1159 T>C, c.573del, and c.1829_1831del were observed in one patient each. The recurrent pathogenic variants (c.1426C>T and c.1160G>A) were observed in patients with ASMD Type B (Table 3).

**TABLE 3:** Genetic, familial findings, and current location of patient.

ASMD subtype	Patient	*SMPD1* genotype	Consanguinity	Positive family history	Sibling relationship	Current province
A	1	—	—	—	—	Catamarca
	2	—	No	No	—	Córdoba
	3	—	No	Yes	Sibling pair (Patient 4)	Córdoba
	4	—	No	Yes	Sibling pair (Patient 3)	Córdoba
B	5	c.1492C>T/c.319‐2A>G	No	No	—	Buenos Aires
	6	—	No	Yes	Sibling pair (Patient 7)	Córdoba
	7	—	No	Yes	Sibling pair (Patient 6)	Córdoba
	8	—	No	No	—	Córdoba
	9	c.1426C>T/c.1160G>A	No	No	—	Chaco
	10	c.1107_1110dup/c.1426C>T	No	No	—	Tucumán
	11	—	—	—	—	Buenos Aires
	12	c.1426C>T/c.1426C>T	—	—	—	Santiago del Estero
	13	c.1327C>T/c.1801G>A	No	No	—	Chaco
	14	c.96G>A/c.1160G>A	No	Yes	Sibling pair (Patient 19)	Río Negro
	15	—	No	No	—	Buenos Aires
	16	c.573del/c.1829_1831del	No	No	—	Buenos Aires
A/B	17	c.1406A>G/c.1553C>T	No	No	—	Salta
	18	c.518dup/c.1159T>C	No	No	—	Santa Fe
—	19	—	No	Yes	Sibling pair (Patient 14)	Río Negro
Total *n*/*N*, %			0/16, 0%	6/16, 38%		

*Note:* – denotes data not available. Shaded gray rows indicate patient is deceased.

Abbreviations: ASMD, acid sphingomyelinase deficiency; *SMPD1*, sphingomyelin phosphodiesterase 1.

Among the 19 patients, 11 were alive at last follow‐up, with ages ranging from early childhood to 73 years. Surviving patients were predominantly those with ASMD Type B and A/B phenotypes and generally exhibited a milder clinical course compared with those who died. They were primarily characterized by visceral involvement, including hepatosplenomegaly and cytopenias, with absent or milder neurological manifestations. All deceased patients (8/8) had documented pulmonary involvement, whereas 4 of the 11 surviving patients did not show evidence of pulmonary disease. Of the deceased patients, 4/8 (50%) had ASMD Type A, 2/8 (25%) had ASMD Type B, 1/8 (13%) had ASMD Type A/B, and the subtype of 1 deceased patient was unknown. The average age of death of the 4 deceased patients with ASMD Type A was 3.0 years (range: 2.2 to 4.3 years). The 2 deceased patients with ASMD Type B died at ages 18 and 68. The deceased patient with Type A/B died at 2.7 years old. The most frequent cause of death was respiratory failure, which occurred in 6 (75%) deceased patients (3 with ASMD Type A, 1 Type B, 1 Type A/B, and 1 of unknown subtype). All patients who died from respiratory failure had documented pulmonary involvement before death as noted above. One patient died of pulmonary hemorrhage (ASMD Type A), and 1 died of unknown causes (Type B).

## 4 Discussion

This retrospective case series describes the clinical presentation of 19 patients diagnosed with ASMD in Argentina from 1988 to 2022. This investigation reveals that diagnosis of ASMD was often significantly delayed, with an average gap of more than 7 years from symptom onset to definitive diagnosis. Hepatosplenomegaly, anemia, and pulmonary involvement were the most common clinical features reported; however, a wide range of clinical features was observed across all ASMD subtypes (Table 2), reflecting the complex and variable spectrum of the disease.

This case series also demonstrated the diagnostic challenges that arise from the heterogeneous clinical presentation of ASMD. A significant majority (79%) of patients were referred to multiple clinical specialties before receiving a diagnosis. The most frequently referred specialties were pediatrics (*n* = 13), hematology (*n* = 11), gastroenterology (*n* = 8), and pulmonology (*n* = 6; Table 1). Visiting multiple specialists with potentially long wait times between appointments adds to patient and family burden and contributes to the substantial delay between symptom onset and diagnosis [7]. The most common initial presenting symptoms observed in this cohort included hepatosplenomegaly, anemia, and globular abdomen, which are nonspecific symptoms that overlap with those of more common conditions, complicating early recognition and diagnosis. Two patients in this cohort were initially misdiagnosed with Gaucher disease (Table 1), a more prevalent lysosomal storage disease resulting from a glucocerebrosidase enzyme deficiency that shares some clinical manifestations with ASMD [12]. The two misdiagnosed patients were siblings who initially presented with hepatosplenomegaly and anemia as adolescents and experienced a gap of over 30 years between the onset of symptoms and receiving the proper diagnosis of ASMD Type B. The older sibling died of respiratory failure at 68 years of age. Effective enzyme replacement therapies are now available for the specific treatment of both ASMD and Gaucher disease, making prompt and accurate diagnosis essential for patients to start an appropriate treatment [13, 14, 15, 16]. In patients exhibiting symptoms of possible lysosomal storage disease, simultaneous determination of ASM and glucocerebrosidase activity is recommended [6].

Substantial variability in symptom onset, age at diagnosis, and disease severity was observed in this patient cohort. Consistent with the known differences in disease severity, none of the four patients with ASMD Type A were alive at the end of the study period, whereas 10/12 (83%) patients with Type B and 1/2 (50%) patients with Type A/B survived. These findings reflect the milder clinical course observed in nonneuronopathic forms of the disease. Neuronopathic involvement is recognized as a differentiating factor between ASMD Type A and B, with neurological defects typically absent or mild in patients with Type B, whereas severe neurodegeneration is often present in patients with Type A [1, 3, 8]. In our cohort, all four patients with ASMD Type A exhibited hypotonia and two showed signs of developmental delay, whereas the two patients with ASMD Type A/B exhibited hypotonia, ataxia, and neurocognitive impairment, with onset of neurological symptoms at 5 months in one patient and at 20 years in the other. Pulmonary involvement, manifested mainly by interstitial lung infiltrates, was observed in nearly 80% of patients, and respiratory failure was the leading cause of death in our case series, underscoring the potential benefits of respiratory monitoring in patients with ASMD [3]. The presence of multisystem involvement highlights the need for a multidisciplinary team approach to the management of ASMD [4, 8].

Although genetic sequencing data were available for only a subset of patients, the distribution of *SMPD1* variants observed in this cohort is broadly consistent with previously reported observations in the literature and in the ClinVar database, although no definitive genotype–phenotype associations could be established given the limited sample size. Variants previously reported to preserve partial ASM activity, including many missense variants (e.g., c.1426C>T [17], c.1492C>T [18], c.1160G>A (ClinVar Variation ID [19] 4061987), and c.1801G>A [20]) and nonframeshift variant c.1829_1831del [21], were identified in patients with ASMD Type B in this cohort. In contrast, the 518dup (ClinVar 189153) frameshift variant—predicted to have a more severe impact on protein function—was observed in a patient with a severe phenotype (hypotonia, ataxia, and neurocognitive impairment). However, not all variants followed this pattern. Several missense variants were found in patients with Type A/B disease (e.g., c.1406A>G [22], c.1553C>T [22], and c.1159T>C [17]). Additionally, several severe variants were present in patients with heterozygous genotypes, specifically c.319‐2A>G (splice variant, ClinVar 2 071 325), c.573del (frameshift) [21, 23], c.1107_1110dup (frameshift, ClinVar 3 754 612), c.96G>A (stop‐gain) [24], and c.1327C>T (stop‐gain) [25], where co‐occurrence with milder variants may have contributed to a less severe clinical presentation.

The autosomal recessive nature of ASMD inheritance makes family screening and genetic counseling highly important for first‐degree relatives of patients with an ASMD diagnosis [3, 4]. This was highlighted by the presence of three pairs of siblings with ASMD in our cohort, including a pair of brothers with Type A who both died before age 5. Although consanguinity can increase the likelihood of ASMD inheritance, no patients in our cohort were born to consanguineous parents. The absence of *SMPD1* variants reported in neighboring countries (e.g., c.1076C>A, found commonly in patients from Chile [26]) may reflect regional founder effects and population‐specific genetic backgrounds, as well as the limited sample size of the cohort.

This case series had several limitations. First, the retrospective nature of the data collection may introduce bias due to incomplete data and variability in the documentation of data. Second, although reflective of the rarity of ASMD, the small sample size of patients included in this cohort limits the generalizability of the findings. Lastly, genetic sequencing data were not available for all patients, which limits analysis of genotype–phenotype correlations.

## 5 Conclusion

This retrospective case series describes a population of patients with ASMD that presented with a broad spectrum of symptoms and experienced significant delays before receiving an accurate diagnosis. Increased awareness of ASMD and its heterogeneous presentation is critical for prompt diagnosis, particularly in patients with unexplained organ dysfunction, anemia, or interstitial lung disease. Physicians who encounter patients with unexplained symptoms, including hepatosplenomegaly, anemia, lung infiltrates, globular abdomen, thrombocytopenia, osteopenia, leukopenia, or cherry‐red spot—with or without neurological involvement—should be prompted to perform a panel for lysosomal enzyme deficiencies, including ASM. Although this study was not designed to assess treatment outcomes, the availability of enzyme replacement therapy can alleviate disease burden. Educating clinicians across specialties, particularly pediatrics, hematology, gastroenterology/hepatology, and pulmonology, is necessary to increase symptom recognition, reduce time to diagnosis and management, and prevent misdiagnosis.

## Author Contributions

Maria Cristina Robin was involved in planning, conducting, writing, and reporting the study. All authors participated in data collection and analysis. All authors contributed to the manuscript and approved the submitted version.

## Funding

This publication was sponsored through a Sanofi Medical Award to help with manuscript preparation and publication. Writing support was provided by Anna Josephson, PhD, of IMPRINT Science, New York, NY, USA, and was funded by Sanofi.

## Ethics Statement

This retrospective case series was reviewed and approved by the Training, Teaching, and Research Committee of the Ministry of Health of the Province of Santiago del Estero, Argentina.

## Consent

Informed written consent was obtained from the patients' legal guardian/next of kin prior to inclusion.

## Conflicts of Interest

The authors disclose that Sanofi provided funding for manuscript preparation and publication, including medical writing support. Sanofi had no role in study design, data collection, data analysis, interpretation of the data, or critical review of the manuscript.

## Supporting information


**Figure S1:** Geographic distribution of the 19 patients included in the study across Argentina. Patients are grouped by province of residence and indicated by labeled callouts.

## Data Availability

The data that support the findings of this study are available from the corresponding author upon reasonable request.
