# Supplementary figures and images for: Clinical Characteristics of 19 Patients With Acid Sphingomyelinase Deficiency: A Case Series From Multiple Centers in Argentina
Source: JIMD Rep. 2026 Jun 28;67(4):e70104. doi: 10.1002/jmd2.70104 (PMC13310959; doi:10.1002/jmd2.70104)

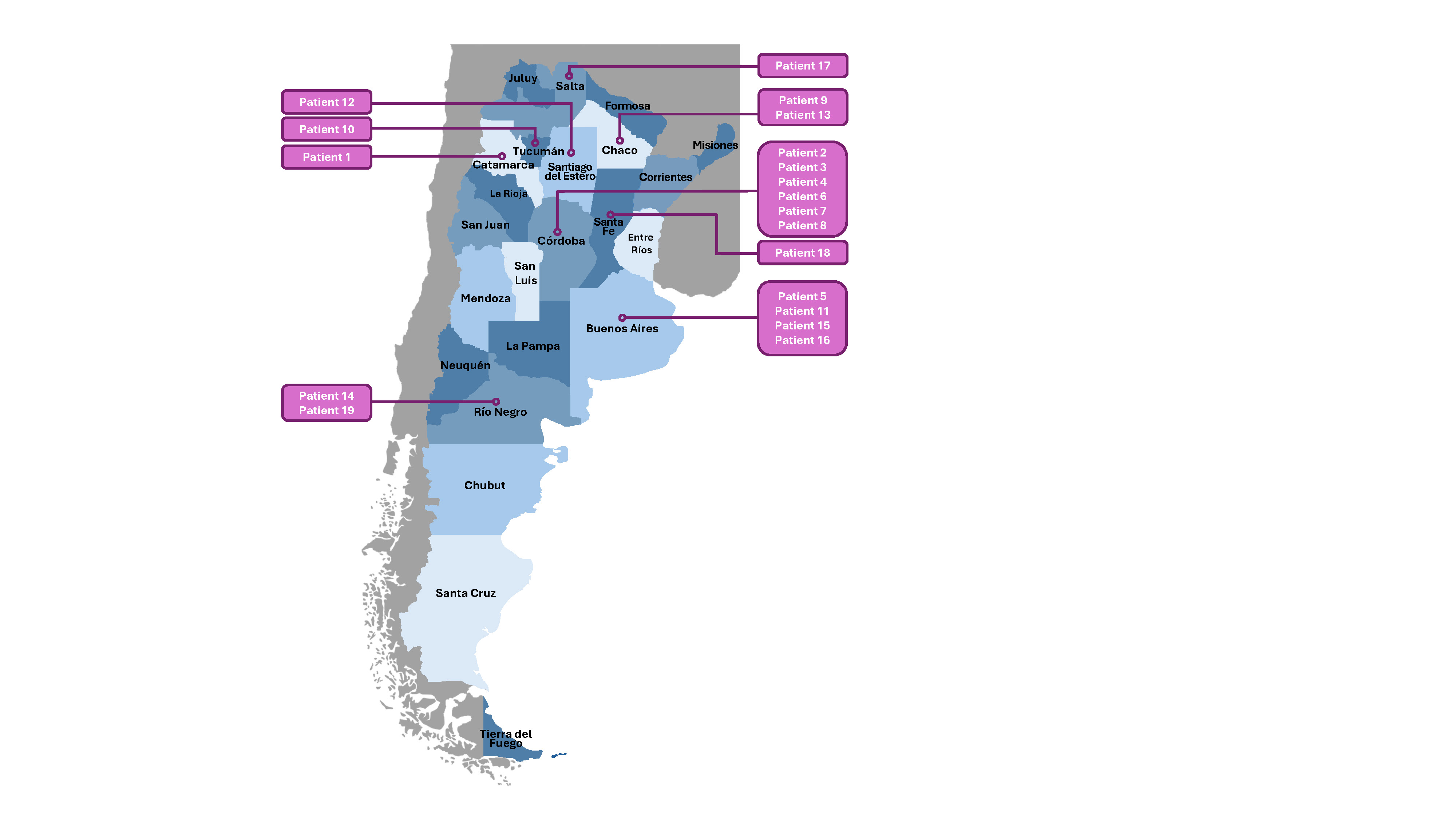

Supplement: Supplementary file 1 — Figure S1: Geographic distribution of the 19 patients included in the study across Argentina. Patients are grouped by province of residence and indicated by labeled callouts. [file JMD2-67-e70104-s001.jpg]
